# Imaging-based deep learning in kidney diseases: recent progress and future prospects

**DOI:** 10.1186/s13244-024-01636-5

**Published:** 2024-02-16

**Authors:** Meng Zhang, Zheng Ye, Enyu Yuan, Xinyang Lv, Yiteng Zhang, Yuqi Tan, Chunchao Xia, Jing Tang, Jin Huang, Zhenlin Li

**Affiliations:** 1 Department of Radiology, West China Hospital, Sichuan University, No. 37 Guoxue Alley, Chengdu, 610041 China; 2 Medical Equipment Innovation Research Center, West China Hospital, Sichuan University, No. 37 Guoxue Alley, Chengdu, 610041 China; 3 Med+X Center for Manufacturing, West China Hospital, Sichuan University, No. 37 Guoxue Alley, Chengdu, 610041 China

**Keywords:** Kidney diseases, Renal tumor, Non-neoplastic renal disease, Deep learning, Medical imaging

## Abstract

Kidney diseases result from various causes, which can generally be divided into neoplastic and non-neoplastic diseases. Deep learning based on medical imaging is an established methodology for further data mining and an evolving field of expertise, which provides the possibility for precise management of kidney diseases. Recently, imaging-based deep learning has been widely applied to many clinical scenarios of kidney diseases including organ segmentation, lesion detection, differential diagnosis, surgical planning, and prognosis prediction, which can provide support for disease diagnosis and management. In this review, we will introduce the basic methodology of imaging-based deep learning and its recent clinical applications in neoplastic and non-neoplastic kidney diseases. Additionally, we further discuss its current challenges and future prospects and conclude that achieving data balance, addressing heterogeneity, and managing data size remain challenges for imaging-based deep learning. Meanwhile, the interpretability of algorithms, ethical risks, and barriers of bias assessment are also issues that require consideration in future development. We hope to provide urologists, nephrologists, and radiologists with clear ideas about imaging-based deep learning and reveal its great potential in clinical practice.

**Critical relevance statement** The wide clinical applications of imaging-based deep learning in kidney diseases can help doctors to diagnose, treat, and manage patients with neoplastic or non-neoplastic renal diseases.

**Key points**

• Imaging-based deep learning is widely applied to neoplastic and non-neoplastic renal diseases.

• Imaging-based deep learning improves the accuracy of the delineation, diagnosis, and evaluation of kidney diseases.

• The small dataset, various lesion sizes, and so on are still challenges for deep learning.

**Graphical Abstract:**

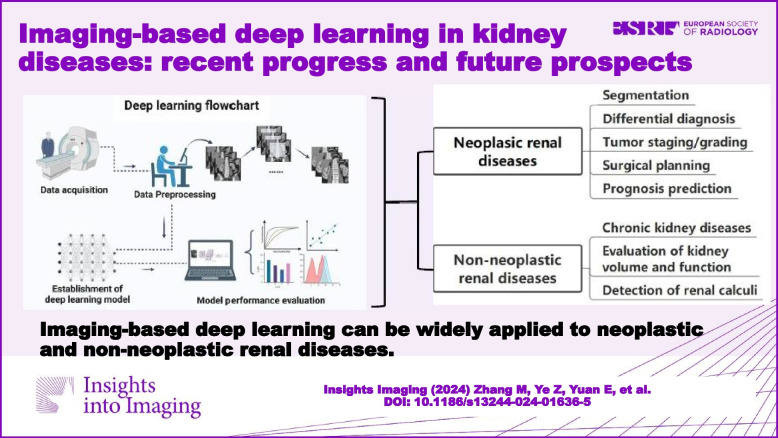

## Introduction

The kidneys are essential organs responsible for filtering waste products from the blood, regulating electrolytes and fluid balance, and producing hormones to balance blood pressure and red blood cell production [1–5]. Kidney diseases can result from various causes, including diabetes, hypertension, autoimmune diseases, infections, and genetic disorders [1, 4, 6], which can generally be divided into neoplastic and non-neoplastic renal diseases. It is worth noting that renal cell carcinoma accounts for a large proportion of neoplastic kidney diseases, and its incidence is on the rise [7], posing great harm to human health. In addition, the non-neoplastic kidney diseases, such as chronic kidney diseases and end-stage kidney failure, can be problematic health burdens for patients and even life-threatening.

Medical imaging as a non-invasive method, including ultrasound (US), computed tomography (CT), magnetic resonance imaging (MRI), and nuclear medicine, plays a critical role in the diagnosis and management of kidney diseases. These imaging methods provide clinicians with valuable information about the renal structure and function, including details about renal blood flow and tissue characteristics, which can help in accurate diagnosis and guide the treatment of kidney diseases [8–11]. However, the conventional interpretation of medical images can be time-consuming and prone to errors, especially in complex cases such as kidney cancer or chronic kidney disease [9, 12, 13]. In recent years, deep learning algorithms have been proposed to mine data and combine information to perform more detailed analysis [14, 15], showing great potential in improving diagnostic accuracy and efficiency of medical image analysis in kidney diseases.

Deep learning is an evolving field of artificial intelligence that uses artificial neural networks to model complex relationships between input data and output predictions. The convolutional neural network (CNN) allows deep learning models to automatically learn discriminative features and patterns that are difficult to detect using traditional image analysis methods [16]. Moreover, deep learning models can be trained on large datasets of labeled medical images to achieve high accuracy and robustness in various clinical scenarios. In kidney diseases, imaging-based deep learning methods have been applied to various medical imaging modalities, such as CT, MRI, US, and single-photon emission computed tomography (SPECT). These methods have been used for a wide range of tasks, including renal tumor applications (e.g., tumor segmentation, differential diagnosis, tumor staging, and grading) and non-neoplastic renal diseases [17–24].

Most of the previous reviews in this field focused on the methodology of deep learning or the discussions on kidney cancer rather than kidney diseases in general [25–29]. Therefore, we hope to summarize the clinical applications of imaging-based deep learning in kidney diseases as comprehensively as possible, so as to provide urologists, nephrologists, and radiologists with clear ideas about this approach and reveal its great potential in clinical settings. This review will firstly introduce the methodology of imaging-based deep learning, summarize its recent clinical applications in neoplastic and non-neoplastic kidney diseases, and finally discuss its challenges and possibilities in future development.

## Methodology of deep learning

**Fig. 1:**
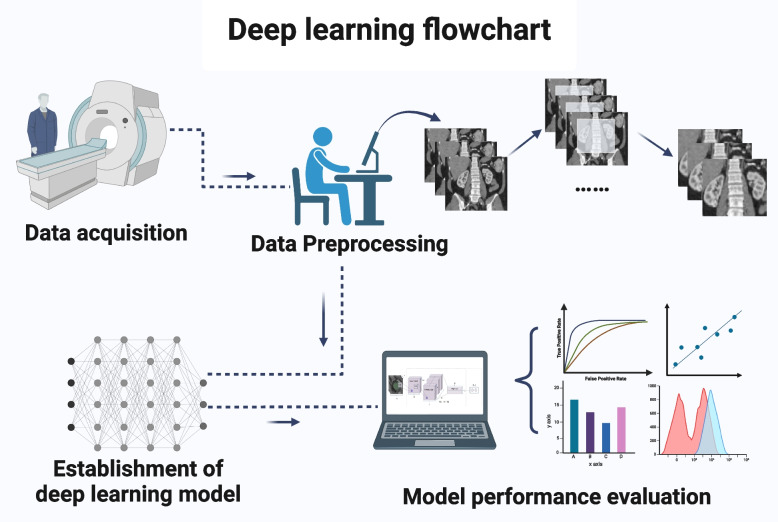
Deep learning flowchart. The methodology of deep learning consists of the following steps, data acquisition, data preprocessing, establishment of deep learning model, and model performance evaluation

The methodology of deep learning in kidney disease involves a series of steps that are critical to the development, training, and deployment of deep learning models for clinical practice (Fig. 1). These steps include data acquisition, preprocessing, model selection, training, validation, and testing, as well as the evaluation and interpretation of the model results. In this section, we will provide a detailed overview of each step.

### Data acquisition

The first step in deep learning for kidney diseases is data acquisition. In order to develop a deep learning model, a large dataset of medical images is usually needed. The quality of the data used for training is also crucial to the accuracy and generality of the algorithm [30, 31]. Multi-source data (e.g., different modalities, different machines, and different imaging parameters) can decrease the bias introduced by the data collection but may also increase the model’s inability to converge [32–35]. The model developers should have a clear understanding of where the model is intended to work and collect data in this domain as much as possible. This step lays the foundation for the subsequent steps in the deep learning process.

### Data preprocessing

The second step is data preprocessing, which involves cleaning and preparing the dataset for use in deep learning models. This step is essential to improve the quality of the data and avoid the effect on the accuracy of the algorithm. There are several techniques used in data preprocessing, including noise reduction, image normalization, and image registration [22, 36–38].

Another important aspect of data preprocessing is data augmentation. Data augmentation generates several slightly modified copies of existing data, by means like rotation, scaling, and cropping, to reduce overfitting when training models [39]. It is worth mentioning that generative adversarial networks can utilize different contrasts and modalities of existing imaging protocols to generate new synthetic images with high authenticity [40–44], demonstrating great potential in data augmentation.

What is more, data preprocessing also involves data labeling. The labels are what we expect the model to output, and they come in different forms depending on the type of learning task. For instance, in renal tumor classification [12, 19, 20, 45], data labeling requires assigning a class or category to each image in the dataset. In addition, matrices or tensors denoting the target area are usually used as labels in renal tumor segmentation [17, 46]. However, for the generative model, no additional label is needed, which belongs to self-supervised learning.

### Establishment of deep learning model

The next step is to establish the deep learning model, including model architecture selection, model training, and hyperparameter adjustment by model validation. There are different kinds of deep learning architectures that can be used for medical image analysis, and most of them are built from the CNN. The CNN, initially designed to process images, is an important adaptation of the Multilayer perceptron which is the simplest deep neural network [47]. Besides, model architecture selection is the process of choosing the appropriate deep learning architecture according to the different learning tasks. For example, U-Net and its 3D variants are often used for the segmentation task [35–37, 46], while residual network (ResNet) and its variants are usually used for the classification task [20].

Once the model architecture is selected, it needs to be trained. This involves feeding preprocessed training data into the deep learning algorithm and adjusting the weights of the model to minimize the difference between the predicted output and actual output. Meanwhile, hyperparameters, which are variables that control the behavior of the model, are also the key components in the algorithm, such as learning rate, epoch, batch size, and number of layers. After model training, the hyperparameters need to be adjusted by model validation to optimize the performance of the training model [48]. Through continuous model training and hyperparameters adjustment, the optimal model, including the optimal weights and the optimal hyperparameters, is established by comparison.

### Evaluation of model performance

Evaluation is an important step in the deep learning process to assess the performance of the developed model on a separate testing set. The evaluation of deep learning models in kidney diseases includes several metrics, such as accuracy, precision, F1-score [49, 50], area under the receiver operating characteristic curve (AUROC) [51], Dice similarity coefficient, and Jaccard similarity coefficient [52].

The choice of these metrics depends on the specific problem being addressed. For instance, in classification tasks, accuracy, sensitivity, and specificity are commonly used metrics. While the Dice similarity coefficient and Jaccard similarity coefficient are often used as the evaluation metrics in segmentation tasks. The model performance evaluation helps to ensure that the model has not simply memorized the training data and can accurately generalize to new data.

## Applications of deep learning in kidney diseases

Imaging-based deep learning has been widely applied to kidney diseases, including renal tumors and non-neoplastic renal diseases. We have reviewed the relevant studies and summarized their clinical applications as follows.

### Renal tumor

#### Segmentation

Accurate segmentation of regions of interest is the basis of quantitative image analysis and essential in the research of imaging-based deep learning. Manual segmentation is time-consuming and labor-intensive. In addition, the accuracy and reproducibility of manual or semi-automatic segmentation heavily depends on the experience of the annotator and the complexity of the images. Therefore, much research is devoted to developing automatic segmentation algorithms, aiming to provide end-to-end segmentation methods to greatly save the cost of the process.

**Fig. 2:**
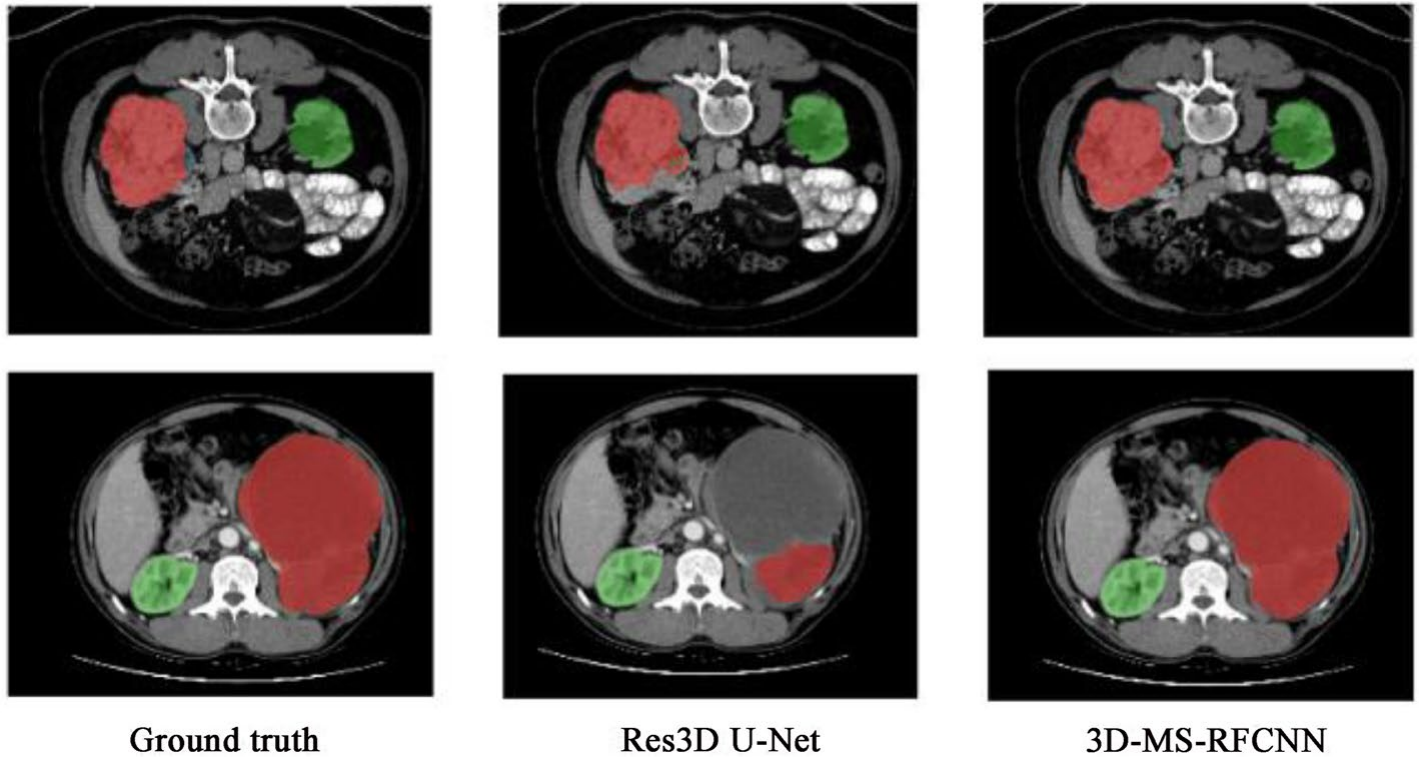
Examples of representative results of kidney tumor segmentation on internal (KiTS) and external (in-house) validation datasets: Images in the first row are examples of an extremely large tumor from the internal validation dataset. Images in the second row are examples of an extremely large tumor from the external validation dataset. The green color indicates the kidney, and the red color indicates the tumor. Image reproduced with permission from "Yang E, et al. (2022) 3D multi-scale residual fully convolutional neural network for segmentation of extremely large-sized kidney tumor. Comput Methods Programs Biomed 215:106,616."

Yang et al. [53] proposed a 3D Multi-Scale Residual Fully Convolutional Neural Network for segmenting kidney tumors larger than 7 cm. The method employed a multi-scale approach to capture global contextual features, demonstrating greater accuracy than the state-of-the-art method, with a Dice score of 0.9390 for the Kidney and Kidney Tumor Segmentation Challenge dataset and 0.8575 for the in-house hospital dataset (Fig. 2). The study suggests that the proposed network can be useful in the field of medical image analysis for accurately segmenting and analyzing large-sized kidney tumors. However, the segmentation performance of small and medium-sized tumors still needs to be further improved, and more efficient deep learning models need to be used to provide more focal information to avoid over-segmentation.

In addition to the simple segmentation of tumor boundaries, the accurate segmentation of renal tumors and renal structures is also crucial. The Meta Greyscale Adaptive Network was proposed for 3D integrated renal structure segmentation in CT angiography (CTA) images [17]. The proposed model segmented the kidneys, renal tumors, arteries, and veins in one inference, addressing challenges such as low contrast and network representation preferences caused by grayscale distribution variation inter-images. The study enrolled 123 patients and achieved an average Dice coefficient of 87.9% for renal structures, showing promising results for renal cancer treatment.

#### Differential diagnosis

Deep learning provides a feasible approach to precisely identify the imaging features of renal mass, so as to differentiate and diagnose them. Zabihollahy et al. [12] recently employed a deep learning model developed on contrast-enhanced computed tomography (CECT) images from 155 patients to differentiate renal cell carcinoma (RCC) from benign solid renal masses (renal oncocytomas and fat-poor renal angiomyolipoma) and validated the model on CECT images from 160 patients. The proposed CNN algorithm adopted the semi-automated method and the accuracy, precision, and recall rate obtained were 83.75%, 89.05%, and 91.73%, respectively.

The oncocytoma and chromophobe RCC (ChRCC) are similar radiologically and immunohistochemically, making it difficult to distinguish between them. Baghdadi et al. [54] developed a semi-automated CT-based deep learning system to compute the tumor-to-cortex peak early-phase enhancement ratio which was then used to identify benign renal oncocytoma and ChRCC. The accuracy, sensitivity, and specificity obtained in the internal validation were 95%, 100%, and 89%, respectively, which outperformed manual diagnosis. Pedersen et al. [20] also proposed a deep learning approach, which uses more than 20,000 2D CT images, to classify the two types of tumors. And the model was tested on three independent datasets with 90.0–97.7% accuracy when evaluated image by image, and up to 100% accuracy when evaluated by 51% majority vote of individual image classifications for each patient.

These renal tumor identification studies further suggest that deep learning has the potential to solve a similar problem from multiple dimensions, and its possibility in clinical application needs to be explored. Meanwhile, it is of great reference value that the classification results at the patient level are determined by those at the two-dimensional level in the differential diagnosis.

#### Tumor staging/grading

**Fig. 3:**
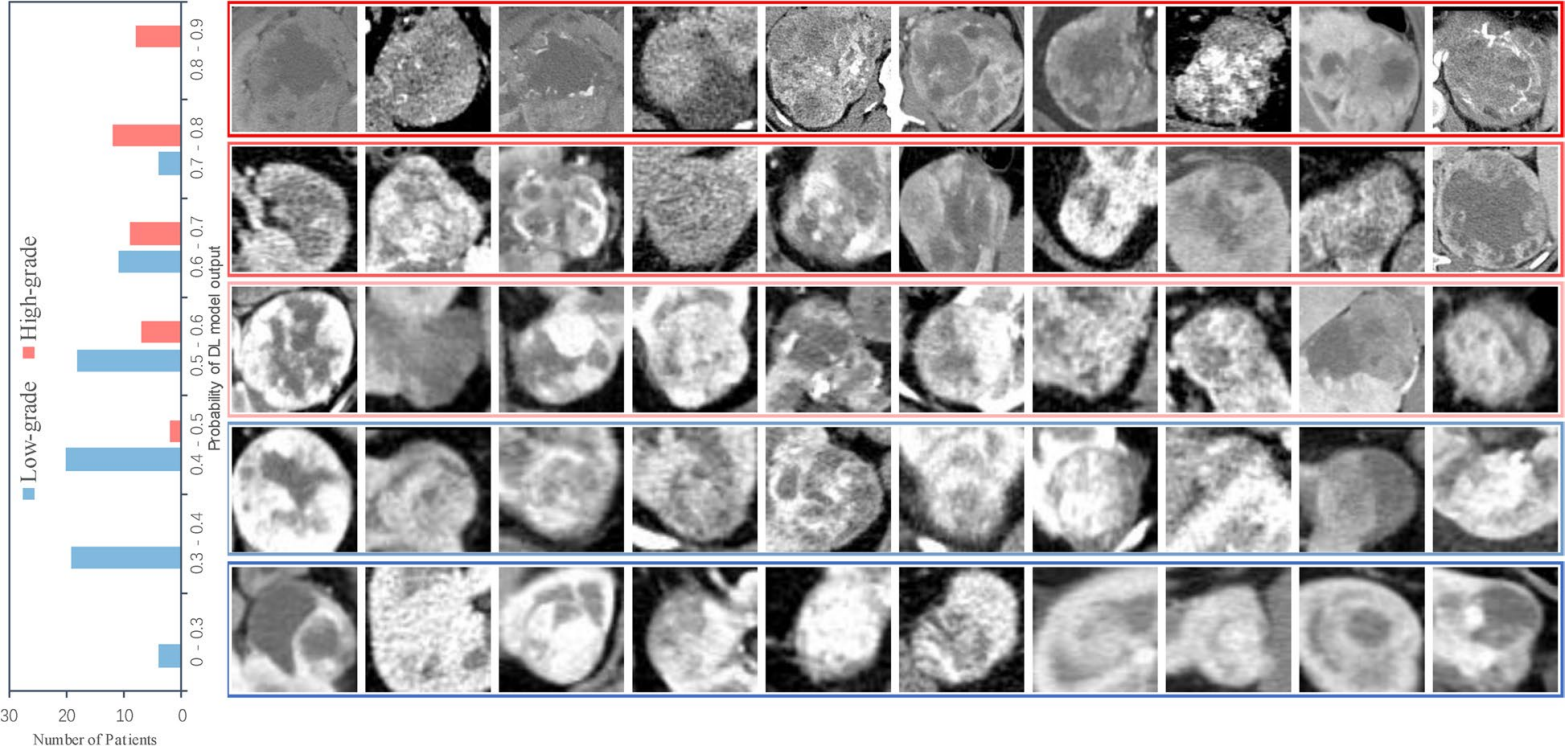
Network output probabilities for low-grade and high-grade patients. The left subplot is the network output probability distribution of low-grade and high-grade patients. The right subplot is the CT images of low-grade and high-grade patients with different network output probabilities. "Xu L, et al. (2022) Deep Learning Using CT Images to Grade Clear Cell Renal Cell Carcinoma: Development and Validation of a Prediction Model. Cancers 14: 2574."

For renal tumors, especially malignancies, identification of tumor stage and grade is crucial for monitoring the patient’s condition and developing individualized treatment strategies. In a recent retrospective study, Xu et al. developed four different types of deep learning models to grade clear cell renal cell carcinoma (ccRCC) by learning CT images [55], and all achieved satisfactory performance in external validation (Fig. 3). Meanwhile, Xu et al. further proposed an innovative weight calculation method to weigh the outputs of the four models and subsequently combine them to obtain the final grading decision. This behavior was like the expert consultation that multiple experts analyzed the patient’s condition and combined the opinions with each other so as to make the judgment more precise and reliable. Finally, the ensemble model enhanced the network generalization ability and further improved the predictive performance with an AUROC of 0.882. The study avoids the problem of a small correlation of image contents between the pre-training and developing process in deep learning research and is meaningful for the research with label noise and category imbalance.

In addition, Zhao et al. aimed to evaluate the efficacy of a ResNet in differentiating low-grade (grade I–II) from high-grade (grade III–IV) in stage I and II RCC using routine MRI [56]. The study included 376 patients with 430 RCC lesions, of which 353 were classified by Fuhrman grading for model training, validating, and testing, and 77 by WHO/ISUP grading as a separate test set. The final deep learning model achieved a test accuracy of 0.88, sensitivity of 0.89, and specificity of 0.88 in the Fuhrman test set, and a test accuracy of 0.83, sensitivity of 0.92, and specificity of 0.78 in the WHO/ISUP test set. However, the heterogeneity of the different data acquisition parameters at different institutions is still a barrier to expanding research dataset. And the automatic segmentation for small-size kidney tumor is still challenging even though automatic segmentation has shown great performance in other organs.

Moreover, Hussain et al. proposed a deep neural network based on learnable image histogram, which can learn the complex and subtle task-specific textural features from original CT images, focusing not only on RCC grading but also on RCC staging [21]. For RCC Fuhrman grading, the method which was developing on the CT dataset from 159 patients obtained the estimated accuracy of 80%. What is more, Hussain et al. have also tried to compare the RCC grading performance of their approach with a wide range of methods including the traditional machine learning approaches. The performances of these conventional machine learning are lower than that of the proposed method. As for TNM staging, the researchers divided the data into stage low (I–II) and stage high (III–IV) for RCC staging, and the estimated accuracy was 83%. These results suggest that deep learning has great potential in predicting RCC aggressiveness and malignant degree, showing that deep learning is non-invasive and efficient in tumor grading and staging, and the future application of this method in clinical scenario is foreseeable.

#### Surgical planning

At present, fine segmentation of renal arteries on abdominal CTA images and accurate assessment of vascular structure especially small vessels are critical to the diagnosis and treatment of renal tumors, and they are the essential steps of preoperative planning in both open surgery and minimal-invasive surgery.

**Fig. 4:**
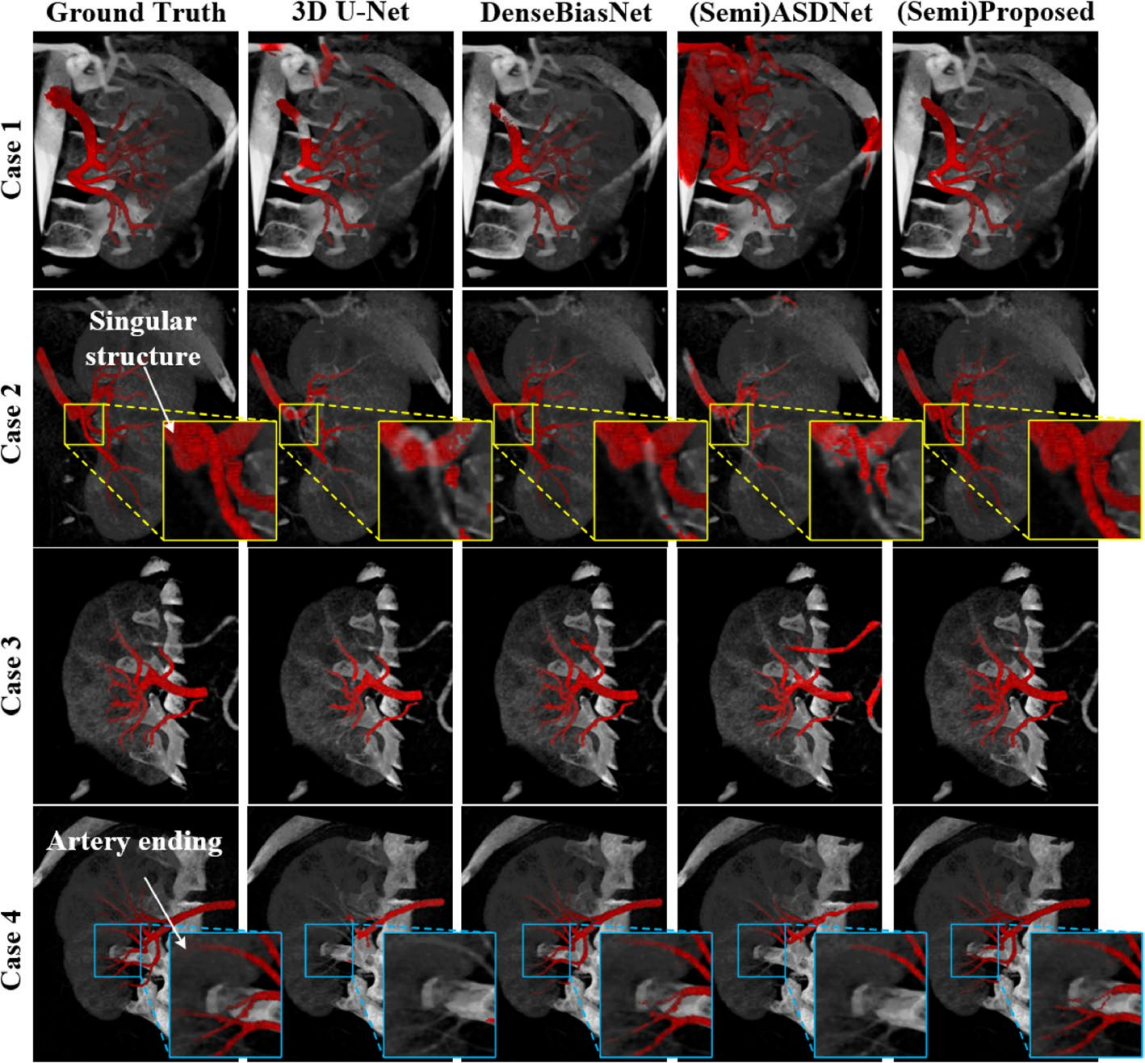
The visual superiority of the proposed framework. The blue boxes indicate the high segmentation quality of artery endings via the framework and the yellow boxes indicate the high segmentation quality of the singular regions brought by the author’s deep priori anatomy (DPA) strategy (For interpretation of the references to color in this figure legend). Image reproduced with permission from "He Y, et al. (2020) Dense biased networks with deep priori anatomy and hard region adaptation: Semi-supervised learning for fine renal artery segmentation. Med Image Anal 63:101,722."

In a recent study, Wang et al. proposed a new approach for precisely estimating the renal vascular dominant region using a Voronoi diagram, combining a neural network and tensor-based graph-cut methods for kidney and renal artery segmentation [57]. The accuracy of kidney segmentation in 27 cases reached a Dice score of 95% and the accuracy of renal artery segmentation in 8 cases obtained a centerline overlap ratio of 80%. The final dominant-region estimation accuracy achieved a Dice coefficient of 80%. However, even though the results of kidney segmentation are optimistic, the different renal pathology patterns and image slice thickness still have negative effects on the performance. Moreover, a novel 3D semi-supervised framework developed by He et al. for fine renal artery segmentation also achieved similar results with the Dice coefficient of 88.4% [58]. Figure 4 illustrated the visual advantages of this model, the framework was capable to achieve the good performance for the 3D fine renal artery segmentation and the high quality for the singular structure segmentation so as to help the clinicians locate the blood supply area precisely, and consequently improved the efficiency of renal preoperative planning and reduced the cost of individualized treatment in clinical practice.

Deep learning frameworks show the great potential in the real clinical surgical environment, which can meet the demand of fine renal arteries and thin structures segmentation and obtain relatively high quality, and help doctors accurately and directly locate and evaluate vascular structures, formulate precise surgical plan, create greater survival opportunities and higher quality of life for patients.

#### Prognosis prediction

Prognosis prediction is also crucial for the management of kidney tumor patients during treatment. Prognosis is related to complex medical data, such as clinical risk factors, histopathological patterns, and image manifestations. Integrating these heterogenous multi-modal data poses challenges in data analysis. The development of deep learning models provides ideas for multi-modal data analysis and can further mine the data to make more accurate judgments.

For instance, there is a study that aimed to develop and evaluate a multimodal deep learning model (MMDLM) for prognosis prediction in ccRCC patients [59]. The MMDLM was trained on multiscale histopathological images, CT/MRI scans, and genomic data from whole exome sequencing of 230 patients in The Cancer Genome Atlas cohort and 18 patients in the Mainz cohort. The MMDLM showed great performance in predicting the 5-year survival state of ccRCC patients, with a mean Harrell’s concordance index of 0.7791 and a mean accuracy of 83.43%. The findings suggest that MMDLM can contribute to prognosis prediction and potentially improve the clinical management of ccRCC. However, missing the comparison with other clinical tools which include more comprehensive clinical data makes the superiority of MMDLM hardly ascertainable. And because of the small external validation dataset, its generalizability also needs additional studies to prove.

### Non-neoplastic renal diseases

In addition to the wide application of imaging-based deep learning in renal tumors, several recent studies have reported that deep learning also focuses on the clinical application of non-neoplastic renal diseases, including the distinction of healthy kidneys from kidneys with chronic kidney disease (CKD), the evaluation of kidney function, and the detection of renal calculi.

#### Chronic kidney diseases

With the application of deep learning in kidney diseases, the mining of kidney image data is deepened, and the characteristic changes are also used to further improve the accuracy of CKD diagnosis. Recently, Lee et al. conducted a multi-task study using deep learning, which included 909 patients (385 with CKD and 524 without CKD), and their kidney US images were used for analysis to detect CKD [51]. The Mask regional convolutional neural network model was used in the kidney and liver segmentation, from which measurable features such as kidney length and kidney-to-liver echogenicity ratio were extracted. Concomitantly, the ResNet-18 was used for CKD diagnosis. It was worth noting that three models were proposed. The average AUROC of the first model achieved a level of 0.81 (sensitivity 78.2%, specificity 71.5%, and accuracy 74.4%), which only used the US image information for analysis. The second model added the extracted measurable features on the basis of the first model, and the average AUROC improved to 0.88 (sensitivity 86.1%, specificity 77.5%, and accuracy 81.2%). The last model, which further incorporated clinical information (e.g., diabetes history), resulted in the average AUROC of 0.91 (sensitivity 89.4%, specificity 82.9%, and accuracy 85.9%).

Many studies applying deep learning to US images have shown lower accuracy than other imaging modalities. However, the AUROC of CKD classification in the above research was significantly improved, indicating that the image measurable features extracted through the deep learning process can be used as supplementary data to refine the model and improve the accuracy. Meanwhile, the developed joint model further took advantage of the clinical information and achieved better results than the single imaging model, demonstrating that relevant clinical features can be appropriately fused to construct multivariate models. Furthermore, the model performance probably be further improved by bringing more comprehensive and actual-clinical data into study, including the patients whose estimated glomerular filtration rate (eGFR) is between 60 mL/min/1.73 m^2^ and 90 mL/min/1.73 m^2^, and the US images with large cysts, solid masses, and hydronephrosis. This suggests that taking the real-world clinical situation into consideration potentially enhances the model practicability.

#### Evaluation of kidney volume and function

In addition, autosomal dominant polycystic kidney disease (ADPKD) results in an increase in total kidney volume (TKV) due to the progressive growth of cysts. Therefore, assessment of TKV is essential for evaluating disease severity, disease progression, and therapeutic response in ADPKD, and the advances in deep learning provide the process more assistance.

Kline et al. [52] established a reference standard TKV based on their previous study and developed a fully automated approach that randomly selected 2000 cases with their MRI data for the training and validation using 400 cases not involved in the training for testing. The model achieved good results on the test dataset with a Jaccard coefficient of 0.94 ± 0.03 and a Dice coefficient of 0.97 ± 0.01 (sensitivity = 0.97 ± 0.02, specificity = 0.99 ± 0.01, and precision = 0.98 ± 0.02). Moreover, van Gastel et al. [60] extended the structure of the network based on the research of Kline et al. and also achieved good results in TKV measurement compared with the conventional manual measurement. These studies revealed the potential of deep learning in TKV measurement, which provided a more time-saving method for disease assessment in patients with ADPKD.

Kuo et al. proposed a deep learning approach for automatically determining the eGFR and CKD status using kidney US images [61]. They used transfer learning and kidney length annotations to develop a neural network that predicts kidney function based on 4505 kidney US images. The model achieved a Pearson correlation coefficient of 0.741 and an overall CKD status classification accuracy of 85.6%. Besides, Pieters et al. [62] also provided a fully automatic method, which was connecting the body-composition parameters extracted from the abdominal CT scans with clinical features to develop the equations estimating creatinine production and obtained the ideal results. These studies above have proved that deep learning can achieve good performance in both renal volume evaluation and kidney function assessment. Meanwhile, the deep learning methods are being improved constantly in routine clinical scenarios and gradually becoming the reliable tool for CKD monitoring and management.

#### Detection of renal calculus

Renal calculi are a common kidney disease and a worldwide health problem, which can be induced by risk factors including obesity, diabetes, hypertension, and metabolic syndrome. The formation of renal calculi can lead to urinary system obstruction, kidney failure, hypertension, CKD, and end-stage renal disease [6, 63]. In clinical practice, the diagnosis of renal calculi generally needs the assistance of imaging techniques, including US and non-contrast-enhanced computed tomography (NCCT). Nowadays, imaging-based deep learning is developing rapidly and is already being used in these techniques to further improve the ability to detect renal calculi.

**Fig. 5:**
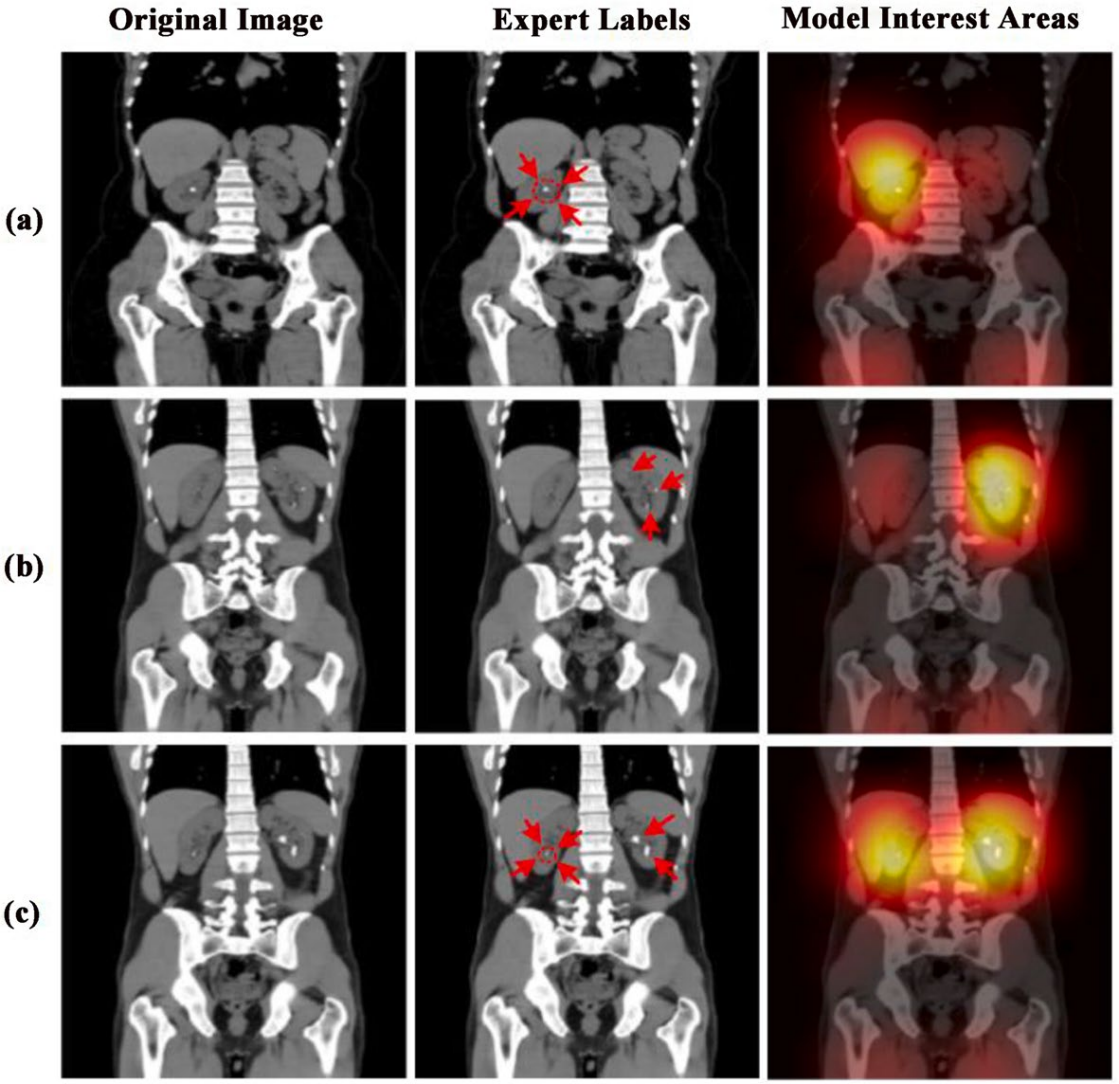
Sample test images showing the areas on which the Deep learning model has concentrated for diagnosis. Red arrows were regions used by experts to show the stones in the images. Image reproduced with permission from "Yildirim K, et al. (2021) Deep learning model for automated kidney stone detection using coronal CT images. Comput Biol Med 135:104,569."

Yildirim et al. [63] developed a coronal CT image-based deep learning model to detect renal calculi on a dataset of 1453 NCCT images and validated the model on an internal cohort of 346 NCCT images (Fig. 5). The proposed cross-residual network model shows excellent performance in detecting renal calculi, and the accuracy, sensitivity, and specificity obtained are 96.82%, 95.76%, and 97%, respectively. At the same time, the study also put forward the proposals to detect kidney stones on axial and sagittal CT images, and the relation between stone size and detection performance could be further explored. However, Caglayan et al. [64] filled the gap, dividing the dataset into three groups based on kidney stone size to evaluate the kidney stone detection performance of the deep learning-assisted model in three imaging axes respectively.

Elton et al. proposed an approach to automatic detection and volumetric segmentation of renal calculi on a dataset of 180 computed tomography colonography scans [65]. The first step of the method was to segment the kidneys, then to combine with the gradient anisotropic diffusion denoising, thresholding, and region growing, and finally classify the detected results as true or false positive, obtaining a sensitivity of 0.86 at 0.5 false positives for each scan and achieving an AUROC of 0.95 on an external validation set for patient-level classification. Meanwhile, there was a good correlation between the model and manual measurements of stone volume. Recently, on the basis of their previous work, Mukherjee et al. proposed a slightly modified pipeline which updated the segmentation method and volume threshold, obtaining a per-scan sensitivity of 97.8% [66]. The renal stone volume and its interval changes on serial CT scans were assessed by the deep learning-based automated measurements and manual measurements respectively, and it also showed good agreement between two kinds of measurements. In these studies, the application of deep learning in the detection and volume measurement of renal calculi is significantly important for the assessment of renal calculus. In addition, future studies that take into account the influence of kidney stone composition may further improve the performance of stone detection and evaluation.

## Current challenges and future prospects

In recent years, numerous studies have demonstrated that medical imaging-based deep learning has become a promising tool to improve the diagnosis and management of kidney diseases, including renal tumor diseases and non-neoplastic renal diseases. However, to maximize its potential, several challenges must be addressed.

One of the main challenges in medical imaging-based deep learning in kidney diseases is the lack of large, diverse datasets [12, 20, 57]. Deep learning algorithms require a significant amount of data to learn and make accurate predictions. However, obtaining large, high-quality datasets of medical images can be challenging, as it requires a collaborative effort from multiple institutions and healthcare providers [67]. This not only involves the intricacies of data collection, but also underscores the ethical need to safeguard patient privacy when sharing data [68]. Meanwhile, dataset balance also needs to be considered, so as to avoid potential bias from data collection. For instance, in renal tumor segmentation tasks [53, 56], the tumor size ranges from small to large, and possibly because of the imbalanced dataset, the model often achieves better segmentation results on some tumors with large size than those with small and medium size. However, the sample reweighting method proposed by Xu et al. may be useful for future study on category imbalance, which effectively balances the contribution of categories with different quantity proportions to the loss function.

Besides, the variability in medical images due to different imaging modalities and protocols is another challenge. For example, medical images of the kidney can be acquired using various imaging modalities [32], including CT, MRI, and US, each with its own strengths and limitations. Moreover, it is mentioned in several studies above that the variation in acquisition parameters and the operators could lead to the data inhomogeneity to affect the performance of the model, emphasizing the importance of data preprocessing and data augmentation [20, 56, 57]. In addition, transfer learning to use the parameters of existing models to initialize new models and train them on new data, and ensemble learning to combine the learning results of multiple independent models may also be the solutions to this problem [69].

Additionally, while deep learning models have shown excellent performance in many clinical applications, understanding how they arrived at a particular prediction is still challenging in clinical practice [67]. This is due to the opacity of deep learning process, which makes it difficult to elucidate the causal relationship between the input and output, consequently giving rise to ethical risks. When doctors employ deep learning algorithms for clinical decisions, if there are mistakes, the lack of algorithm interpretability and relevant regulations makes it difficult to clearly identify the accountable party [70]. Simultaneously, achieving a clear definition of the predictors in decision-making becomes unattainable when evaluating the risk of bias, creating an impediment in bias assessment [71]. Therefore, many researchers now attempt to validate the established model on a larger scale in external centers [63–65]. If the same satisfactory results can be obtained as the internal validation, the impact of poor interpretability on clinicians can be reduced to a certain extent. Moreover, some advanced techniques are also attempting to simulate the mathematical relationships between adjacent pixels to provide an interpretable framework for deep learning. Topological data analysis (TDA) is a newer paradigm that can extract information on the shape of data and can be combined with a technique known as persistent homology to transform data into visually meaningful representations, breaking the black-box process of traditional deep learning model to some extent [72]. With the use of TDA, researchers have made remarkable progress in the understanding (pathophysiological features, etiology, prognosis) of several diseases, including cancer, asthma, and chronic lung disorders. However, further research is needed to contribute to the interpretability of deep learning and to develop normative reporting guidelines and powerful risk of bias tool on this basis.

Despite the current challenges of medical imaging-based deep learning in kidney disease, its future is still greatly promising. From the above research, it can be seen that medical imaging-based deep learning has the potential to improve the accuracy and efficiency of the diagnosis of renal tumor diseases, including RCC, the most common type of renal tumor. Additionally, medical imaging-based deep learning also has the potential to detect or predict non-neoplastic renal diseases, such as CKD, at an earlier stage, allowing for earlier intervention and better patient outcomes [51]. These models can analyze medical images and identify subtle changes in the renal structure and function that may indicate the presence of non-neoplastic renal diseases, leading to better patient outcomes and reduced healthcare costs. Predictably, deep learning based on medical imaging has the potential to provide personalized medicine for patients with kidney diseases. Medical imaging-based deep learning models can provide a more accurate diagnosis based on the individual unique characteristics, such as tumor/lesion size, location, and histological subtype, to help predict the progression of kidney diseases and develop personalized treatment plans for patients.

## Conclusion

Recent research provides evidence that imaging-based deep learning has great potential in data mining and analysis and has been widely applied in the field of kidney diseases. Both in the clinical scenarios of renal tumors and non-tumor diseases, promising performance has been demonstrated. However, the promotion of deep learning in clinical practice is still facing challenges due to the poor interpretability and repeatability of deep learning, which is also related to the fact that current studies on deep learning tend to focus on specific clinical situations. Therefore, there are still many areas to be improved before deep learning can effectively serve clinical work.

## Data Availability

Not applicable.

## Abbreviations

ADPKD: Autosomal dominant polycystic kidney disease
AUROC: Area under the receiver operating characteristic curve
ccRCC: Clear cell renal cell carcinoma
CECT: Contrast-enhanced computed tomography
ChRCC: Chromophobe renal cell carcinoma
CKD: Chronic kidney disease
CNN: Convolutional neural network
CTA: Computed tomography angiography
eGFR: Estimated glomerular filtration rate
MMDLM: Multi-modal deep learning model
NCCT: Non-contrast-enhanced computed tomography
RCC: Renal cell carcinoma
ResNet: Residual network
TDA: Topological data analysis
TKV: Total kidney volume
